# Knowledge mapping of anaplastic thyroid cancer treatments: a bibliometric analysis (2000-2023)

**DOI:** 10.3389/fonc.2024.1330030

**Published:** 2024-02-14

**Authors:** Shiqiang Liu, Xingmin Yan, Yalong Yang, Yun Xia, Panshi Zhang

**Affiliations:** ^1^ Department of Thyroid and Breast Surgery, Tongji Hospital, Huazhong University of Science and Technology, Wuhan, China; ^2^ Department of Breast Surgery, Hubei Cancer Hospital, Tongji Medical College, Huazhong University of Science and Technology, Wuhan, Hubei, China

**Keywords:** anaplastic thyroid cancer, treatment, bibliometric analysis, endocrine, thyroid cancer

## Abstract

**Context:**

Anaplastic thyroid cancer (ATC) is a relatively rare and extensively malignant kind of thyroid carcinoma. The poor prognosis and high mortality rate of ATC can be attributed to its invasive features and undifferentiated phenotype. At present, there is a lack of efficacious therapeutic options. In light of the elevated fatality rate, it is vital to possess a comprehensive comprehension of the scientific terrain pertaining to ATC. To gather the perspectives of different researchers about the topic of ATC treatment, we did a bibliometric network analysis, which offers a comprehensive view of the scholarly literature.

**Methodology:**

A systematic search was conducted on the WoSCC database to identify publications pertaining to ATC treatment between the years 2000 and 2023. In this bibliometric investigation, the tools VOSviewers, CiteSpace, and the R package “bibliometrix” were employed to investigate the general attributes, developmental framework, and academic frontiers of the subject matter.

**Results:**

1223 publications in total, written by 6937 scholars from 53 areas and 1402 institutions and published in 358 scholarly journals, were analyzed. There has been a gradual increase in the quantity of publications pertaining to ATC treatment. The United States and China emerged as the most prominent nations. The University of Texas MD Anderson Cancer Center and Memorial Sloan Kettering Cancer Counseling Center are prominent research institutions in highly productive countries. The journal *Thyroid* holds a prominent position within its discipline, being widely recognized as both the most popular and highly co-cited publication. According to the available data, Maria Cabanillas has authored the highest number of published articles, while RC Smallridge has received the highest number of co-citations. It turned out that the prevailing keywords encompassed expression, therapy, apoptosis, survival, activation, proliferation, metastasis, and other related terms. Immunotherapy, targeted therapy, and prognostic factors are the emerging research hotspots and trends.

**Conclusions:**

This paper presents a complete overview of research trends and advancements in the treatment of ATC using bibliometric analysis. The acquisition of information will offer vital insights for funding and potential creative strategies in researching the treatment of ATC, which indicates the research frontiers as well as prevalent directions in recent years.

## 1 Introduction

As one of the most prevalent endocrine malignancy in humans, thyroid cancer constitutes approximately 3% of all malignancies reported each year around the world (1). The incidence of thyroid carcinoma continues to rise globally. Based on the histological characteristics, thyroid malignancies can be categorized into four basic classifications: papillary thyroid cancer (PTC), follicular thyroid cancer (FTC), medullary thyroid cancer (MTC) as well as anaplastic thyroid cancer (ATC). The most prevalent type of thyroid cancer, differentiated thyroid carcinoma (DTC) which includes PTC and FTC, responds well to the standard treatment (surgery followed by either radioactive iodine or observation and endocrine therapy) in the majority of patients (2). Differentiated thyroid carcinomas, accounting for approximately 95% of all cases, typically have favorable prognoses with 10-year survival rates reaching 90%-95% (3). As opposed to this, ATC is inevitably lethal. Meanwhile, this rapidly developing initial tumors make total removal impractical to solve (4). In addition, there are currently fewer therapeutic options available for ATC than for DTC, and these usually entail a multimodal strategy (5). The bulk of thyroid cancer-related fatalities are triggered by ATC, which makes up 1-2% of all malignancies of the thyroid and is a rare subtype with the most aggressive biological behavior (6). Even though varied multimodal treatment is carried out, the median overall survival (OS) for ATC is roughly four months from the time of diagnosis, and the disease-specific mortality rate is nearly 100%. These tumors tend to be highly proliferative, typically exhibit higher tumor mutational burden (TMB) than DTC (7, 8). ATC tends to be a challenge to manage considering their aggressive characteristics and undifferentiated phenotype, thereby rendering tumors resistant to traditional therapy (9). Given the high rate of mortality, it is essential to have an in-depth comprehension of scientific landscapes of ATC.

Bibliometrics is an approach used to analyze the literature which combines qualitative as well as quantitative information to determine the output and standing of publications in specific research topic of study (10). It could promote investigations by researchers and improve understanding of therapeutic care. With the goal to gather the perspectives among different academics in the subject matter of ATC treatment, we consequently perform a bibliometric network analysis so as to offer a neutral assessment in scientific publications. This analysis attempts to present researchers with understanding about ATC and its management for further studies.

## 2 Materials and methods

### 2.1 Search strategy

**Figure 1:**
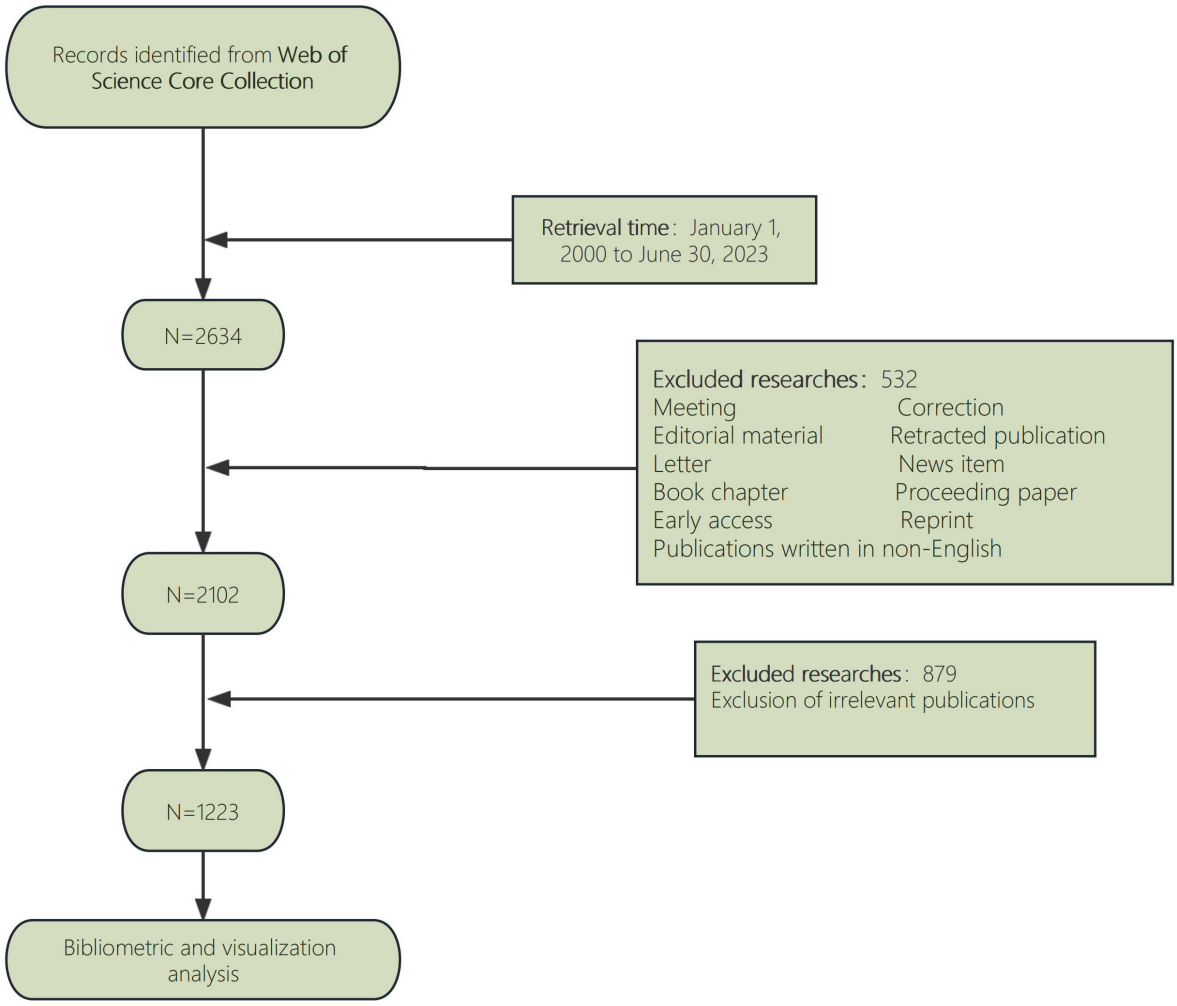
Flow chart for publication’s selection.

A literature search was performed using the Web of Science Core Collection (WoSCC) database on 18 July 2023. And the retrieval strategy was [TS = (“Anaplastic Thyroid Carcinoma” OR “Anaplastic Thyroid Cancer” OR “ATC”)] AND [TS = (“treatment” OR “therapy” OR “Therapeutics”)] AND [article type = (“article” OR “review”)] AND [Time span = (January 1, 2000 to June 30, 2023)] AND LA = [(English)]. In this study, publicly accessible data sets were investigated. Up till June 30, 2023, this search method retrieved 2634 records in English literature. Thus, “original article and review” were established as the inclusion criteria in an effort to reduce bias in our research. Independent researchers with various organizations obtained the raw data and sorted it out to remove papers that weren’t relevant. Eventually, 1223 pieces of literature were collected for an in-depth analysis (**Figure 1**).

### 2.2 Data analysis

The bibliometric analysis and visualization were carried out using VOSviewer, CiteSpace, Excel, and the R package “bibliometrix”. VOSviewer is a bibliometric analysis tool designed to retrieve the essential information from a variety of publications. It is extensively utilized for developing networks for collaboration, co-citation, and co-occurrence (11). In this research, the VOSviewer generally conducted out the analyses listed below: analysis of the nation and the institution, analysis of the author and co-cited authors, journal and co-cited journals and analysis of the co-occurrence of keywords. CiteSpace is capable of being employed to identify collaborations, internal frameworks, major ideas, possible trends, and interactions in the area of study (12). In the present work, CiteSpace was primarily utilized for reference analysis using Citation Bursts and mapping the dual-map overlaying of journals. Using the R package “bibliometrix,” we additionally explored trending topics and developed a worldwide distribution network of publications relating to ATC treatment. Excel was applied to analyze the publications. Furthermore, Journal Citation Reports 2023 was used to identify the category and impact factor of journals quantitatively.

## 3 Results

### 3.1 Publication tendency

**Figure 2:**
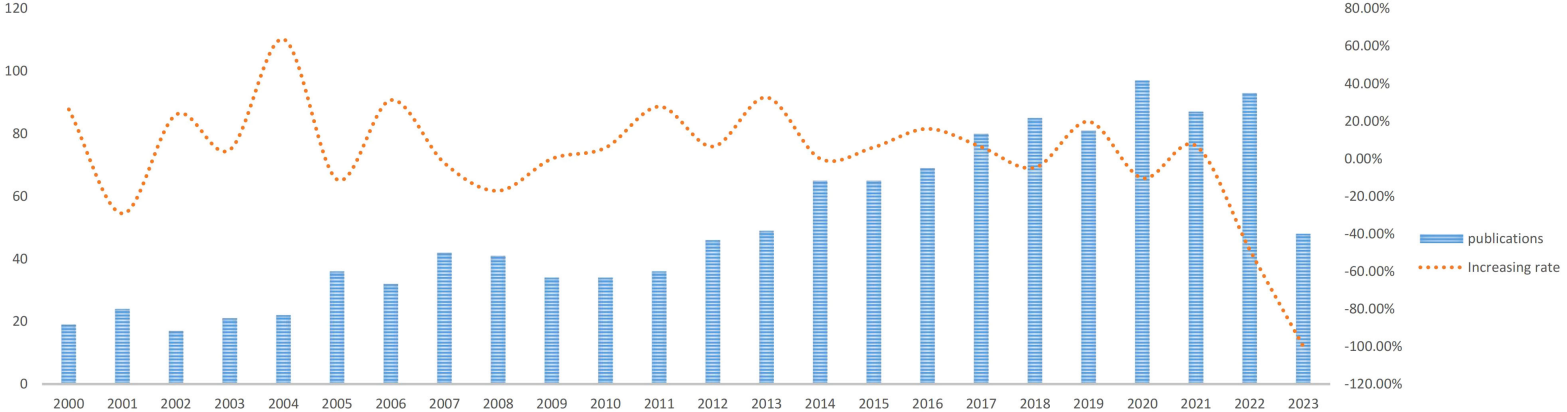
Trends of ATC treatment publications.

There are 1223 articles concerning the treatment of ATC from January 1, 2000 to June 30, 2023. As depicted in **Figure 2**, there were not many publications on ATC treatment research in the first decade (2000-2010), with only an average of 29 articles per year, indicating that studies on ATC treatment was just in the beginning phases of this field’s development. In the next 12 years, the number of articles on ATC treatment almost doubled. With an average of 69 articles each year from 2011 to 2023, there was a substantial increase in the total amount of publications. We could speculate that research on the treatment of ATC grows more in-depth with the quantity of publications rises.

### 3.2 Country and institutional analysis

These publications were issued by 1402 organizations and 53 nations across the globe. The top 12 countries are distributed in Europe (n = 5), North America (n = 4) and Asia (n = 3) (**Table 1**). The United States topped the list of these nations and institutions that conducted research on ATC treatment with 408 publications, followed by China (272 publications), Italy (150 publications), South Korea (121 publications) and Japan (112 publications). More than half of the total (55.6%) of articles came from China and the United States combined. The top 12 institutions are spread over four nations, with half of them in the United States. The University of Texas MD Anderson Cancer Center (n = 47), Memorial Sloan Kettering Cancer Counseling Center (n = 41), Mayo Clinic (n = 36), National Cancer Institute (n = 27), and University of Pisa (n = 26) were the top five organizations with the greatest number of publications.

**Table 1:** Top 12 countries and institutions on research of ATC treatment.

Rank	Country	Counts	Institution	Counts
1	USA (North America)	408	University of Texas MD Anderson Cancer Center	47
2	China (Asia)	272	Memorial Sloan Kettering Counseling Center	41
3	Italy (North America)	150	Mayo Clinic	36
4	South Korea (Asia)	121	National Cancer Institute	27
5	Japan (Asia)	112	University of Pisa	26
6	Germany (Europe)	81	Seoul National University	25
7	France (Europe)	37	Harvard university	24
8	Canada (North America)	31	University of Wisconsin	21
9	UK (Europe)	24	Shanghai Jiao Tong University	20
10	Spain (North America)	23	University of Naples Federico II	20
11	Netherlands (Europe)	17	Yonsei University	20
12	Sweden (Europe)	17	Sapienza University of Rome	19

**Figure 3:**
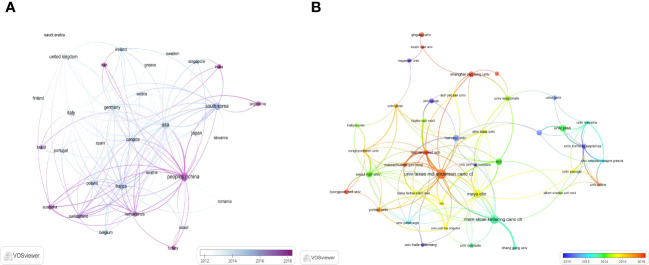
The distribution of countries and institutions publishing research on ATC Treatment. **(A)** Visualization of countries. **(B)** Visualization of institutions.

**Figure 4:**
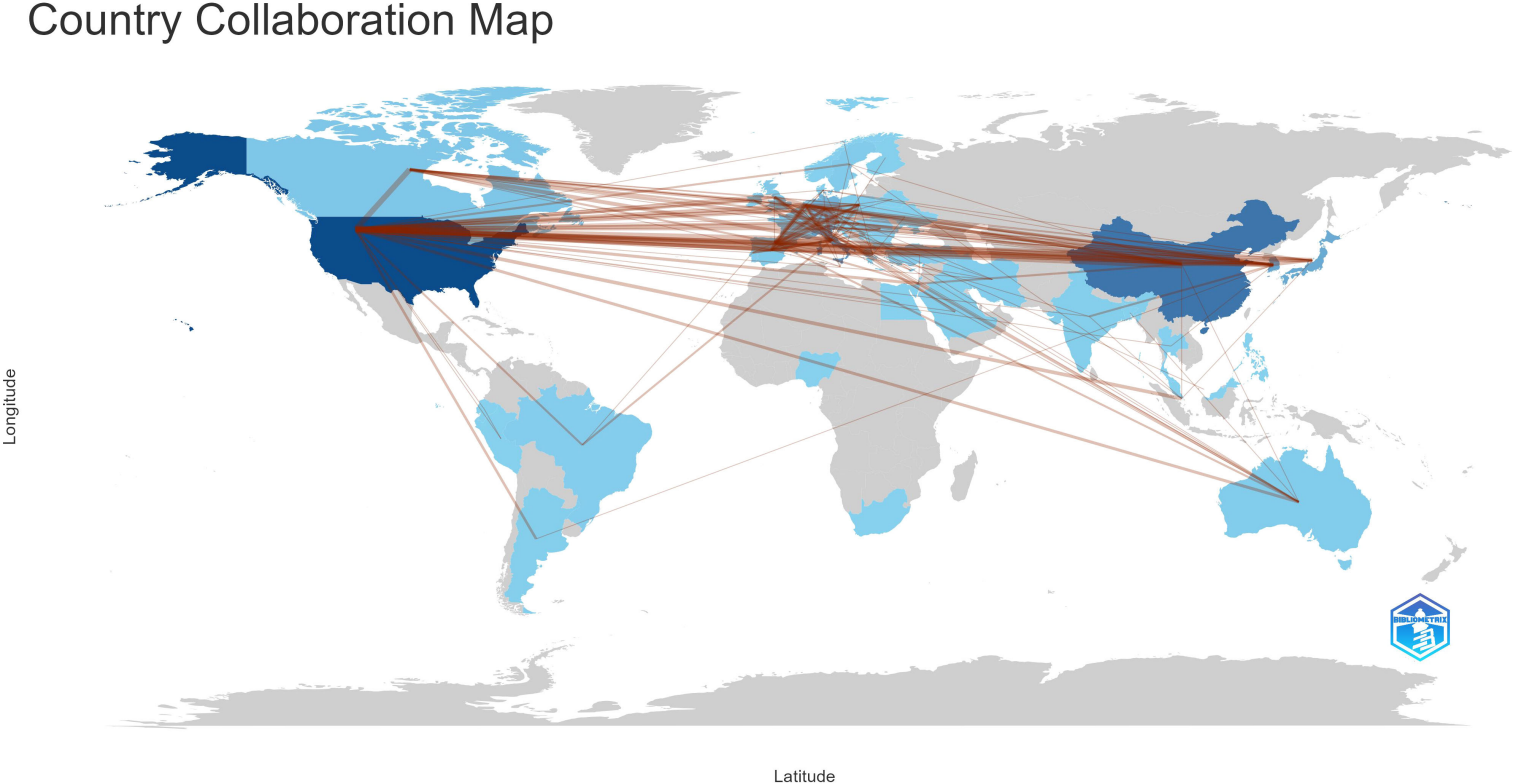
Country collaboration map.

With the use of VOSviewer and Bibliometrix, globally distributed country and institutional distribution networks of publications were mapped. In accordance with the quantity and connections between articles in each nation and organization, we proceeded to develop a collaborative network. Evidently, there is a great deal of active cooperation between many nations. China, for instance, collaborated closely with Netherlands and United States. The USA worked actively with Germany, Canada and Korea (**Figures 3A**, **4**). The University of Texas MD Anderson Cancer Center, Memorial Sloan Kettering Cancer Counseling Center and Mayo Clinic cooperated very tightly on this topic, in line with the network map of the institution’s publications, and they were also the top three institutions with the greatest number of publications. Furthermore, we observed that there was rare communication between Shanghai Jiao Tong University and Seoul National University, the two institutions in East Asia with the greatest the number of publications, while institutions in the North American region had very good interactions with each other (**Figure 3B**).

### 3.3 Journal distribution analysis

ATC treatment-related articles were published in 358 journals. *Thyroid* published the most publications with 88 articles, followed by *Journal of Clinical Endocrinology & Metabolism* with 63 articles, *Endocrine-related Cancer* with 37 articles, and *Clinical Cancer Research* with 29 articles (**Table 2**). Impact factors for these journals in 2023 varied from 3.7 to 11.5. The top 10 journals were presented below, with *Clinical Cancer Research* having the highest impact factor (IF = 11.5) and *Endocrine* having the lowest impact factor (IF = 3.7). Moreover, *Oncotarget* has no impact factor at the moment.

**Table 2:** Top 10 most productive journals and co-cited journals for ATC treatments.

Rank	Journal	Publications	2023 IF	2023 Q	Co-cited Journal	Co-citation	2023 IF	2023 Q
1	Thyroid	88	6.6	Q1	Thyroid	2347	6.6	Q1
2	Journal of Clinical Endocrinology & Metabolism	63	5.8	Q1	Journal of Clinical Endocrinology & Metabolism	2105	5.8	Q1
3	Endocrine-related Cancer	37	3.9	Q2	Cancer Research	1614	11.2	Q1
4	Clinical Cancer Research	29	11.5	Q1	Clinical Cancer Research	1066	11.5	Q1
5	Oncotarget	24	NA	NA	Cancer	1032	6.2	Q2
6	Endocrine	23	3.7	Q3	Oncogene	796	7.5	Q1
7	Plos One	17	3.9	Q2	Endocrine-related Cancer	733	3.9	Q2
8	Cancers	20	5.2	Q1	Journal of Clinical Oncology	771	45.3	Q1
9	International Journal of Molecular Science	18	5.6	Q1	Proceedings of The National Academy of Sciences of The United States of America	662	11.1	Q1
10	Oncology reports	17	4.2	Q2	New England Journal of Medicine	582	158.5	Q1

Additionally, the top 10 co-cited journals were all cited more than 500 times, with *Thyroid* (2347 citations) and *Journal of Clinical Endocrinology & Metabolism* (2105 citations) being the most cited, closely followed by *Cancer Research* (1614 times) and *Clinical Cancer Research* (1066 times). The *New England Journal of Medicine* has the highest impact factor (IF = 158.5) among them, followed by the *Journal of Clinical Oncology* (IF = 45.3). The top two journals with the most citations and co-citations are, coincidentally, *Thyroid* and *Journal of Clinical Endocrinology & Metabolism*.

**Figure 5:**
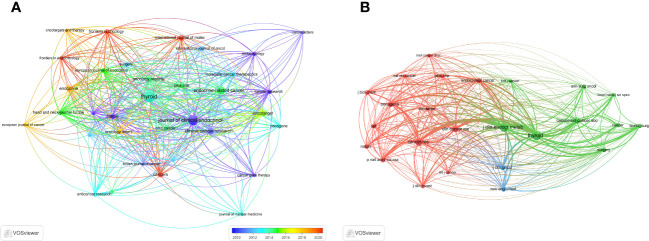
The visualization of journals. **(A)** Journals on research of ATC treatment. **(B)** Co-cited journals.

**Figure 6:**
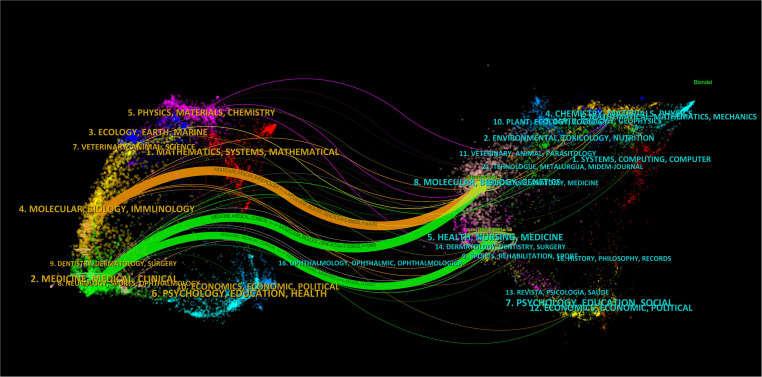
The dual-map overlay of journals on ATC treatment.

We then mapped the journal network after filtering 35 journals based on a minimum of 8 related articles. In the interim, journals with a minimum co-citation of 300 were selected in order to map the co-citation network (**Figures 5A, B**), which exhibited the relationship between journal citations and co-citations. For instance, *Clinical Cancer Research, Endocrine-related Cancer*, and *Thyroid* all had active citation interactions with each other. In addition, *Journal of Clinical Endocrinology & Metabolism* also had active citation relationships with *Oncology reports* and *Molecular cancer therapeutics*. The *Journal of Clinical Endocrinology & Metabolism* also had favorable co-citation associations with publications on *Thyroid*, *Cancer research* and *Endocrine-related cancer*, etc al. The dual-map overlay of journals that we developed with CiteSpace to trace the linkages between cited and cited journals revealed that the major citation pathways were from medicine, clinical, and immunology to molecular, biology, and genetics (**Figure 6**).

### 3.4 Authors and co-cited authors

**Figure 7:**
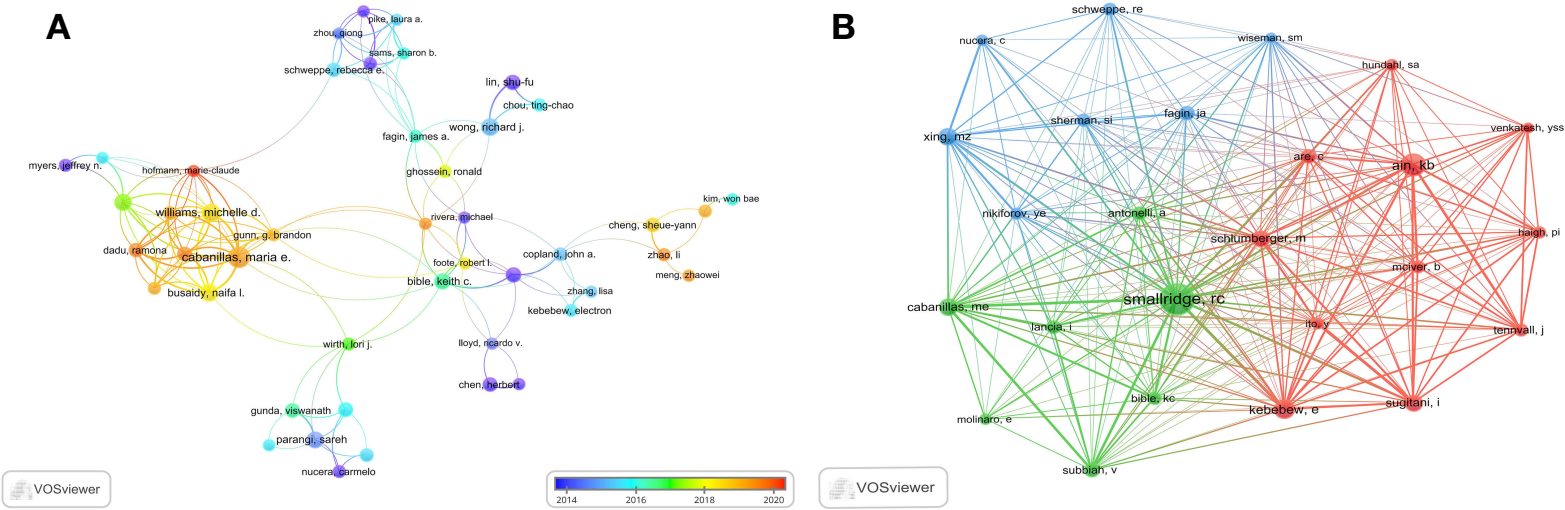
The visualization of authors. **(A)** Authors on research of ATC treatment. **(B)** Co-cited authors.

The researches on ATC treatment involved 6937 authors overall. Cabanillas, Maria (25 papers) led the top ten authors with the most articles published in terms of both publications and citations, followed by Williams Michelle (18 papers) and Wong Richard (16 papers). There were 12 or more articles by each of the top ten authors. Smallridge RC. (469 citations), Ain Kb. (294 citations) and Kebebew E. (218 citations) were the subsequent most-cited authors among the 19354 co-cited authors. In addition, the top ten co-cited authors had received over 140 co-citations in total (**Table 3**). Through VOSviewer, we additionally filtered and visualized the author network based on authors with at least seven published papers and the co-cited network whose authors had at least 90 co-citations (**Figures 7A, B**). It was evident that the creators and co-cited authors actively collaborated.

**Table 3:** Top 10 authors and co-cited authors on research of ATC treatment.

Rank	Authors (publications ≥ 12)	Counts	co-cited authors (Citations ≥ 140)	citations
1	Cabanillas, ME	25	Smallridge, RC	469
2	Williams, Michelle	18	Ain, KB	294
3	Wong, Richard	16	Kebebew, E	218
4	Busaidy, Naifa	16	Cabanillas, ME	198
5	Antonelli, Alessandro	14	Xing, MZ	189
6	Lai, Stephen	14	Schlumberger, M	185
7	Onoda, Naoyoshi	14	Sugitani, I	182
8	Parangi, Sareh	14	Subbiah, V	147
9	Bible, Keith	13	Are, C	143
10	Fallahi, Poupak	12	Fagin, JA	143

### 3.5 Reference distribution analysis

**Figure 8:**
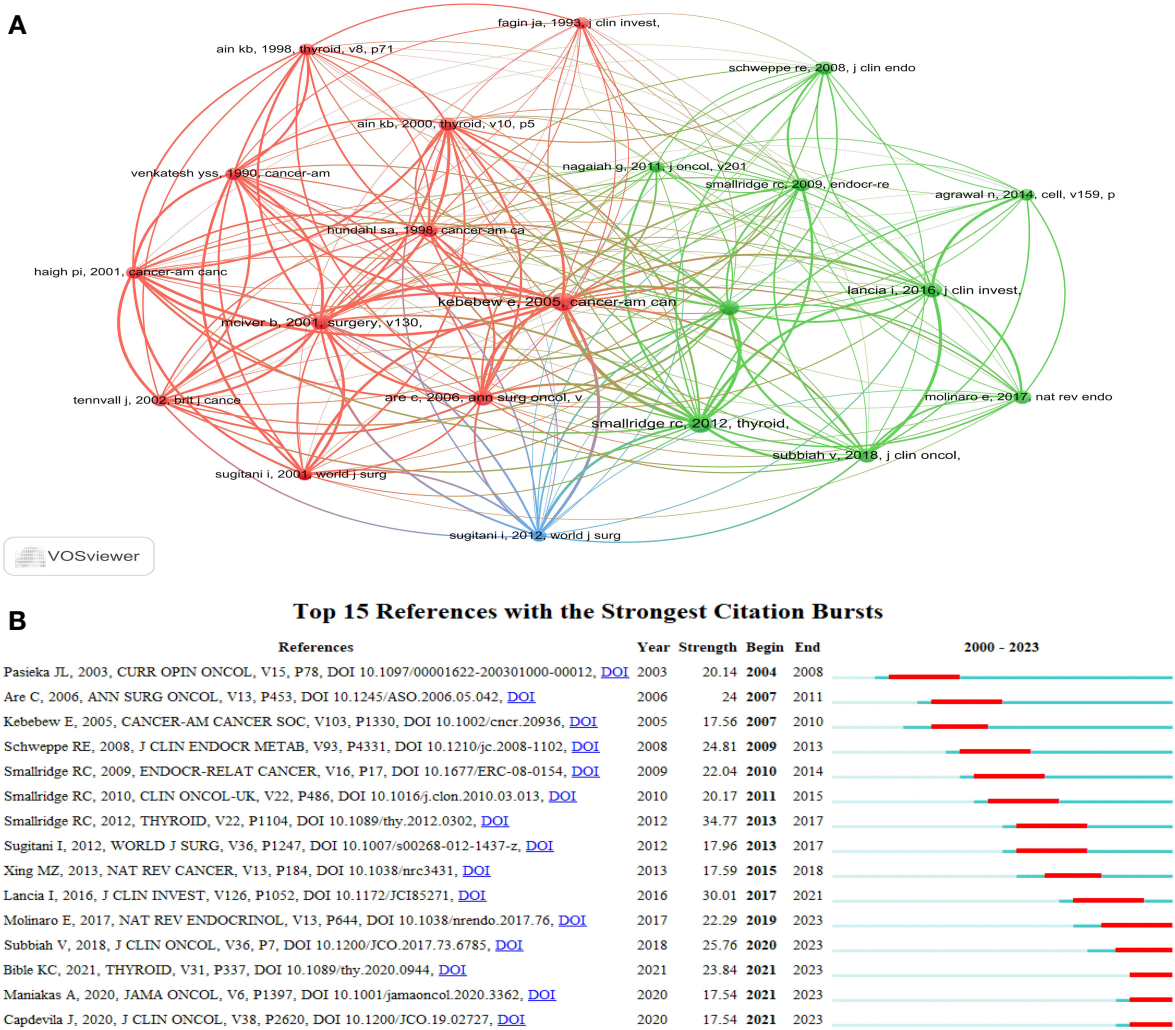
The visualization of references. **(A)** The visualization of co-cited references on research of ATC treatment. **(B)** Top 15 references with strong citation bursts. A red bar indicates high citations in that year.

The top 10 most frequently cited studies are listed in **Table 4**. All of them had more than 98 citations. The piece by Robert C. Smallridge et al. in *Thyroid* from 2012 that was titled “American Thyroid Association Guidelines for Management of Patients with Anaplastic Thyroid Cancer” (13) received the most citations, totaling 188. For the purpose of developing the co-citation network map of the top 21 pieces of research shortlisted, we chose references with co-citations that were equal to or greater than 70 using VOSviewer (**Figure 8A**). In the present research, Bibliometrics identified the top 15 references with high citation bursts, which constituted the sources that frequently received citations by academics in a particular field throughout time. We additionally analyzed and pictured these references (**Figure 8B**). “American Thyroid Association Guidelines for Management of Patients with Anaplastic Thyroid Cancer” by Robert C. Smallridge et al. (2012), published by the journal *Thyroid*, possessed the strongest citation burst, with citation bursts spanning the years 2013 to 2017. “Genetic and transcriptomic hallmarks of poorly differentiated and anaplastic thyroid cancers” (14) by Iñigo Landa et al. was the reference with the second-strongest citation burst (strength = 30.01), with citation bursts across the years 2017 to 2021. These 15 references carried endurance strengths ranging from 2 to 4 years, and their burst strengths typically varied from 17.54 to 34.77.

**Table 4:** Top 10 documents in citation analysis of publications on ATC treatment.

Rank	Title	First author	Corresponding author	Source	Publication year	Total citation
1	American Thyroid Association guidelines for management of patients with anaplastic thyroid cancer	Robert C Smallride	R Michael Tuttle	Thyroid	2012	188
2	Anaplastic thyroid carcinoma. Treatment outcome and prognostic factors	Electron Kebebew	Alex McMillan	Cancer	2005	186
3	Anaplastic thyroid carcinoma: biology, pathogenesis, prognostic factors, and treatment approaches	Chandrakanth Are	Ashok R Shaha	Annals of Surgical Oncology	2006	140
4	Anaplastic thyroid carcinoma: pathogenesis and emerging therapies	RC Smallride	J A Copland	Clinical Oncology	2010	133
5	Genomic and transcriptomic hallmarks of poorly differentiated and anaplastic thyroid cancers	Iñigo Landa	James A Fagin	Journal of Clinical Investigation	2016	130
6	Anaplastic thyroid carcinoma: a 50-year experience at a single institution	B McIver	J R Goellner	Surgery	2001	122
7	Dabrafenib and Trametinib Treatment in Patients with Locally Advanced or Metastatic BRAF V600-Mutant Anaplastic Thyroid Cancer	Vivek Subbiah	Bhumsuk Keam	Journal of Clinical Oncology	2018	114
8	Deoxyribonucleic acid profiling analysis of 40 human thyroid cancer cell lines reveals cross-contamination resulting in cell line redundancy and misidentification	Rebecca E Schweppe	Bryan R Haugen	Journal of Clinical Endocrinology & Metabolism	2008	101
9	Anaplastic thyroid cancer: molecular pathogenesis and emerging therapies	Robert C Smallride	John A Copland	Endocrine-related Cancer	2009	100
10	Treatment of anaplastic thyroid carcinoma with paclitaxel: phase 2 trial using ninety-six-hour infusion. Collaborative Anaplastic Thyroid Cancer Health Intervention Trials (CATCHIT) Group	K B Ain	P A DeSimone	Thyroid	2000	98

### 3.6 Keyword co-occurrence cluster analysis

**Figure 9:**
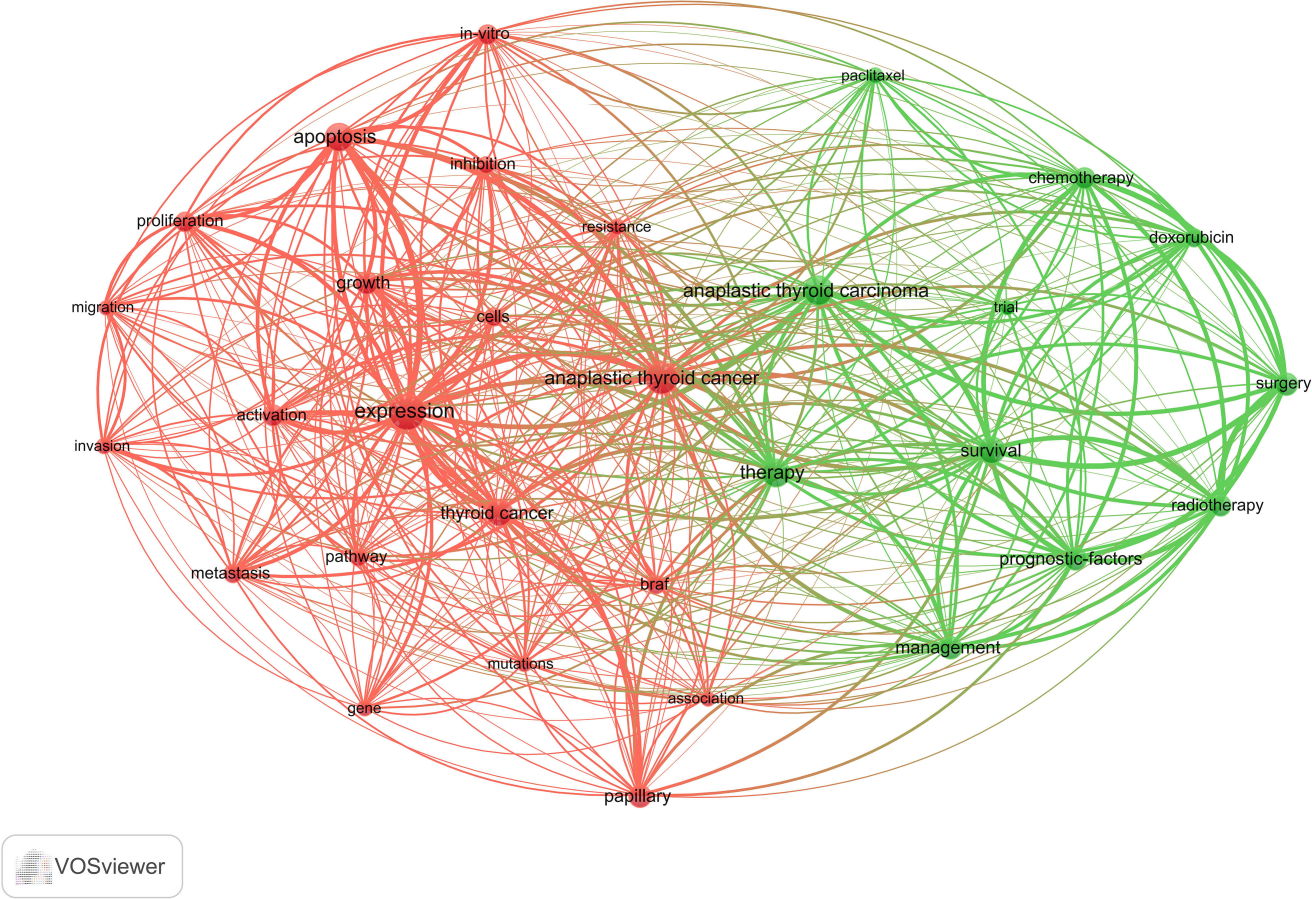
Co-occurrence network of keywords by VOSviewer.

**Figure 10:**
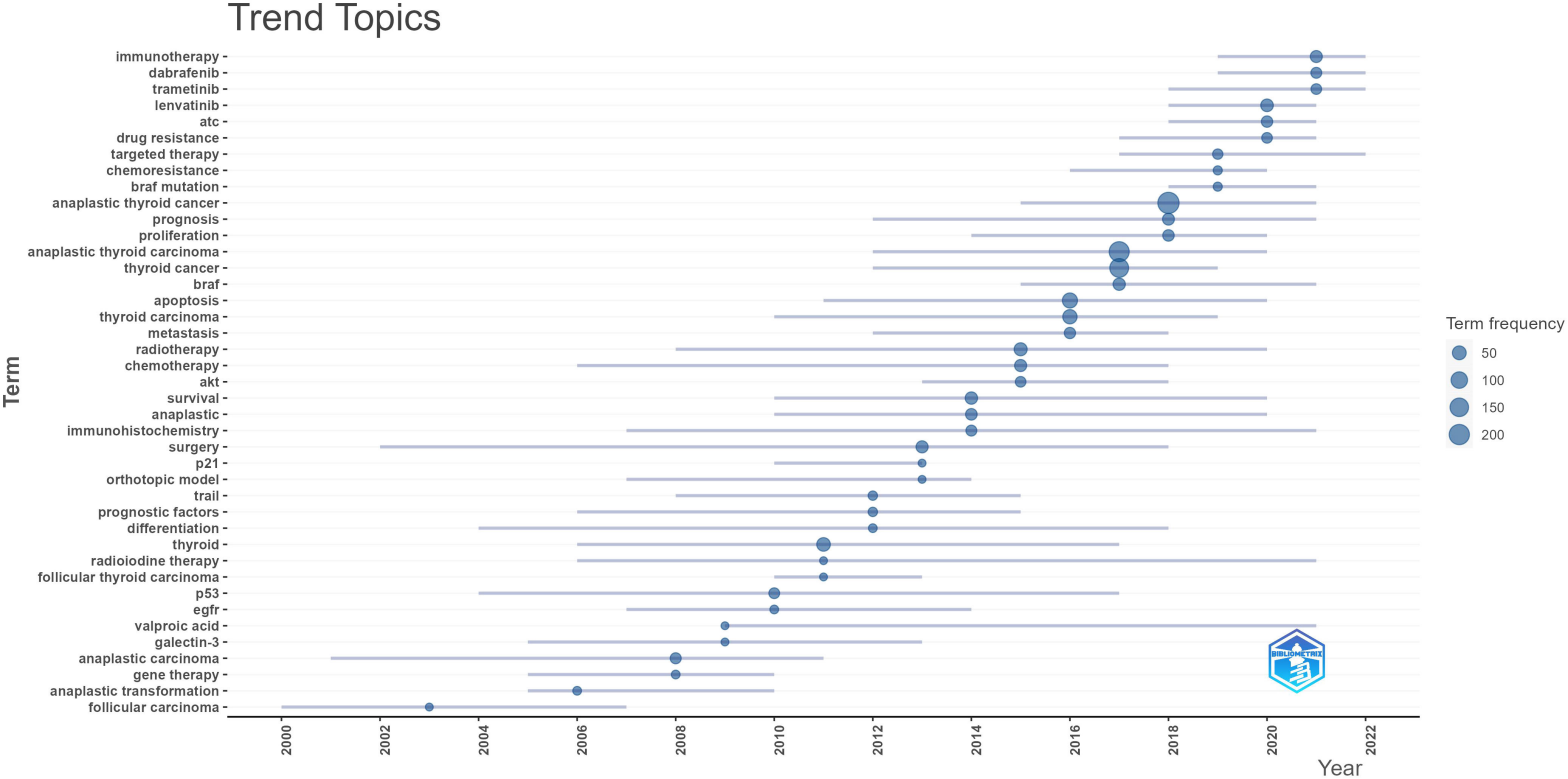
Trend topic by bibliometrics.

The primary concept of a paper is captured through its keywords, and the analysis of keyword co-occurrences is capable of identifying the areas of research that are presently active in a given area. **Table 5** presented a list of the top 20 high-frequency terms that were employed in research on ATC treatment. Expression, which had emerged as the primary area of research for treating ATC, came up 290 times. Through the use of VOSviewer, we screened keywords based on the number of occurrences that met a threshold value of at least 45. Ultimately, a total of 30 keywords had been identified, of which they were primarily clustered into 2 groups of varying hues to indicate different research orientations (**Figure 9**). Management, prognostic-factors, radiation, doxorubicin, paclitaxel, surgery, trial, etc al. were among the keywords clustered in green. Apoptosis, migration, activation, resistance, inhibition, pathway and other phrases comprised the keywords in red clusters. We utilized R package bibliometrix to carry out a trend topic analysis with the goal of better illustrating the different stage hotspots and developmental tracks of ATC treatment (**Figure 10**). According to the keyword trend topic analysis, traditional treatments (such as surgery, chemotherapy, and radiotherapy) and biomarkers were the most prominent topics of research from 2000 to 2015. Immunohistochemistry, treatment, prognostic indicators, P53, P21, EGFR, and other significant terms were the main keywords thereafter. Since 2017, the primary objective of research has switched to targeted therapy, drug resistance, and the proliferation and apoptosis of ATC tumor cells. Simultaneously, immunotherapy has been gaining popularity. The main keywords during this period were *BRAF, BRAF* mutation, drug resistance, chemoresistance, etc al. Drugs such as dabrafenib, trametinib, and lenvatinib were gradually being applied in the field of ATC treatment.

**Table 5:** Top 20 keywords of documents on ATC treatment.

Rank	Keyword	Counts	Rank	Keyword	Counts
1	Expression	290	11	Surgery	106
2	Anaplastic Thyroid Cancer	233	12	Radiotherapy	101
3	Anaplastic Thyroid Carcinoma	194	13	Chemotherapy	94
4	Therapy	179	14	Activation	94
5	Apoptosis	179	15	In-vitro	83
6	Survival	155	16	Proliferation	81
7	Management	117	17	Braf	79
8	Prognostic-factors	113	18	metastasis	74
9	Papillary	111	19	doxorubicin	73
10	Growth	109	20	cells	70

## 4 Discussion

### 4.1 Basic information

Anaplastic thyroid cancer is an extremely lethal form of cancer in humans, accounting for less than 2% of all thyroid carcinomas (15). It is characterized by its aggressive nature, marked by a high death rate, and frequently presents with extrathyroidal extension or metastasis (16). Research on ATC treatment holds significant value in terms of reducing mortality rates and alleviating the financial burden experienced by patients. Historically, the standard treatment modalities encompassed cytotoxic chemotherapy and radiation therapy, either in conjunction with or without surgical intervention. But a significant portion of patients regularly receive palliative or hospice treatment (17). To our knowledge, there is not much research on systematic bibliometric evaluation of scientific publications related to ATC treatment. The aim of the article is to provide an analysis of the present status of the literature on research regarding ATC treatment up until the year 2023. In order to accomplish this objective, bibliometric methodologies were employed to analyze the publication landscape in terms of publication trends, distribution across countries and institutions, journal preferences, reference patterns, keyword usage, and author distribution.

With an average of 29 papers published each year from 2000 to 2010, this area of study remained in its early stages. Between the years 2011 and 2023, there was a notable upward trend in the quantity of published articles, exhibiting a substantial growth. The average yearly publishing rate over this period amounted to 69 papers (**Figure 2**). The field of ATC treatment will advance as publications and studies continue to flourish, which suggests that more academics are becoming interested in related studies. The investigation of prevalent nations yielded the finding that the United States, China, Italy, South Korea, and Japan were identified as the primary five contributing countries, with the United States and China ranking in the top two (**Table 1**). The top three countries with the highest burden of thyroid cancer were China, the United States, and India. Nearly half of the thyroid cancer cases worldwide were found in South and East Asia (18). The prevalence of thyroid malignancies and the level of development in various nations were reflected in the global distribution of ATC publications (19). Some researchers revealed that the incidence rate of the most dangerous anaplastic thyroid carcinoma was not significantly different among areas when compared to other subtypes (20). In the 25 study countries, anaplastic thyroid cancer made up no more than 5% of all thyroid cancers and no more than 2% in 20 of them. The top 12 institutions were dispersed over four different countries, with half of them located in the United States. A strong collaborative partnership existed among certain institutions, including the University of Texas MD Anderson Cancer Center, the Memorial Sloan Kettering Counseling Center, and the Mayo Clinic (**Table 1**, **Figures 3**, **4**). This illustrated how the United States has been leading the field of ATC treatment research. While cooperative partnerships existed in certain nations, the extent and intensity of collaboration among institutions may not meet expectations. There was an inadequate degree of collaboration between institutions in the United States and China. Undoubtedly, this circumstance will impede the advancement of the research domain in the near future.

Most of the literature regarding ATC treatment has been published in publications covering endocrine, cancer, oncology, and clinical research. Among the journals in this discipline, *Thyroid* (IF = 7.561, Q1) stood out as the most extensively read publication, implying that it presently occupied a great deal of popularity among researchers in this particular domain (**Table 2**). Although the highest impact factor belongs to *Clinical Cancer Research*. The majority of the co-cited journals were found to be high-impact Q1 journals. These journals, which are undoubtedly of the highest caliber on a global scale, offer a solid theoretical foundation for upcoming research in ATC treatment.

Taking into consideration the perspective of the author, Cabanillas Maria, an Oncologic Endocrinologist affiliated with the University of Texas MD Anderson Cancer Center in Houston, has made significant contributions to the field with her extensive publications on the subject matter. The main field of her study involved advanced and aggressive forms of thyroid cancer, with a special focus on the utilization of molecularly targeted medicines and immunotherapy (21–25). Over the course of recent decades, notable advancements have been achieved in the development of targeted therapy and immunotherapy. The advancement in gaining an understanding of the disease at a molecular level has facilitated the implementation of novel treatment options, which are now integrated into a multimodal therapeutic approach involving surgery, radiation, and systemic therapy (26). Dr. Cabanillas and her colleagues have directed their attention toward optimizing the procedure for attending to these intricate patients, customizing treatment strategies according to the molecular irregularities present in the tumor, and formulating clinical trials specifically tailored for this particular group of patients. Regarding co-cited writers, Smallridge RC (citation = 469) emerged as the author with the highest frequency of citations, followed by Ain KB (citation = 294) and Kebebew E. (citation = 218). Smallridge RC, a researcher from Mayo Clinic, was currently engaged in the active investigation of multiple potential targets for the treatment of ATC. The research conducted by Smallridge RC, et al. in 2009 demonstrated that the PPAR-gamma agonist, RS5444, had a strong binding affinity and effectively suppressed the proliferation of ATC cells. This inhibition was achieved through the activation of p21 (WAF1/CIP1), a cyclin-dependent kinase inhibitor. Furthermore, the up-regulation of RhoB played a crucial role in the PPAR-gamma-mediated induction of p21, which led to cell cycle arrest (27). The findings of their study revealed that RhoB played a significant role as a new mediator in crucial signaling pathways, indicating an additional potential target for therapeutic strategies in anaplastic thyroid cancer. Over the course of the following year, four ATC cell lines were developed for the purpose of assessing the inhibitory effects of five distinct kinds of medicines on cell growth, particularly in a way dependent on RhoB. In the final analysis, five types of medicines up-regulated RhoB and limited growth dose-responsively, implicating decreased RhoB as a therapeutic target for intervention against ATC (28). Smallridge RC, et al. also actively explored various prospective treatment modalities for anaplastic thyroid cancer, which comprised the combined use of pazopanib and paclitaxel, and their results indicated that the combination of pazopanib and paclitaxel displayed promise as a possible approach to therapy for ATC (29). The researchers also analyzed whether the implementation of intensive multimodal therapy (MMT) at first was correlated with enhanced survival rates for ATC. Moreover, this finding provided additional evidence supporting the notion that MMT may contribute to increased overall survival rates in patients diagnosed with stage IV-A/B ATC (30).

### 4.2 Knowledge base

A reference is co-cited when it is mentioned in more than one other publication. Thus, co-cited references may serve as the foundation for study in a given area (31). In this analysis, we have chosen the top 10 co-cited references that gathered the greatest number of co-citations (**Table 4**). The intention of this selection was to ascertain the foundational research supporting ATC treatment. A guideline written by Robert C. Smallride and other experts and published in *Thyroid* in 2012 was the most frequently cited piece of literature in regards to the topic of ATC treatment (13). This guideline served as the basic foundation for directing ATC treatment in clinical settings. This documentation is the first comprehensive set of guidelines for the management of ATC, offering guidance for addressing this highly aggressive form of malignancy. In cases where a multimodal approach, encompassing surgery, radiation, and systemic therapy, is employed, there is potential for positive outcomes. Additionally, it is worth noting that certain patients with stage IV-B unresectable conditions may exhibit favorable responses to intensive therapeutic interventions. Furthermore, it is worth noting that this rule has undergone revision in the year 2021 (32). The revised version of the guidelines was not among the top 10 documents in the citation analysis of publications on ATC therapy, probably due to the time constraints of this research. The second-most-cited piece of literature in the field of ATC treatment was written by Electron Kebebew and four other researchers and published in 2005 on *Cancer* (33). This work represents an initial investigation into the identification and analysis of prognostic variables associated with anaplastic thyroid carcinoma. The third co-cited study was authored by Ashok R Shaha and Chandrakanth Are, and it was published in the *Annals of Surgical Oncology* in 2006 (34). The author of this review went into detail about the biology, etiology, prognostic variables, and therapeutic approaches associated with ATC. The next research article being co-cited was authored by Iñigo Landa and colleagues, and it was published in the *Journal of Clinical Investigation* in 2016 (14). This publication presented the proof by the scientists that poorly DTC and ATCs originated from well-differentiated tumors as a result of the progressive accumulation of specific genetic defects. The following co-cited articles involved an open-label trial of dabrafenib (a BRAF inhibitor) and trametinib (a MEK inhibitor) combination therapy in *BRAF V600E*-mutated anaplastic thyroid cancer (23). The utilization of dabrafenib and trametinib as therapeutic approaches for anaplastic thyroid carcinoma characterized by the presence of the *BRAF V600E* mutation represented a novel strategy that exhibited good clinical efficacy and favorable tolerability. Broadly speaking, the top 10 co-cited references mostly deal with themes such as biology, etiology, prognostic variables, and exploring therapeutic options. These themes collectively establish a fundamental basis for the given discipline.

### 4.3 Frontiers and hotspots

Citation bursts indicate references that have received a lot of recent citations from other academics and hence signify burgeoning issues within a certain study field (35). Depending on the primary research contents of references with citation bursts (**Table 6**), we can infer that in the field of ATC treatment, the emphasis is primarily on summarizing the experiences of various researchers in this area, exploring potential pathogenesis, and attempting novel treatment approaches (such as novel drug combinations, clinical trials, and immunotherapy). To some extent, the treatment of ATC is still in the exploratory stage.

**Table 6:** Top 15 references with the strongest citation bursts.

Rank	Primary research topic	Strength	Year
1	Anaplastic thyroid cancer	20.14	2003
2	Anaplastic thyroid carcinoma: biology, pathogenesis, prognostic factors, and treatment approaches	24	2006
3	Anaplastic thyroid carcinoma. Treatment outcome and prognostic factors	17.56	2005
4	Deoxyribonucleic acid profiling analysis of 40 human thyroid cancer cell lines reveals cross-contamination resulting in cell line redundancy and misidentification	24.81	2008
5	Anaplastic thyroid cancer: molecular pathogenesis and emerging therapies	22.04	2009
6	Anaplastic thyroid carcinoma: pathogenesis and emerging therapies	20.17	2010
7	American Thyroid Association guidelines for management of patients with anaplastic thyroid cancer	34.77	2012
8	Prognostic factors and treatment outcomes for anaplastic thyroid carcinoma: ATC Research Consortium of Japan cohort study of 677 patients	17.96	2012
9	Molecular pathogenesis and mechanisms of thyroid cancer	17.59	2013
10	Genomic and transcriptomic hallmarks of poorly differentiated and anaplastic thyroid cancers	30.01	2016
11	Anaplastic thyroid carcinoma: from clinicopathology to genetics and advanced therapies	22.29	2017
12	Dabrafenib and Trametinib Treatment in Patients with Locally Advanced or Metastatic BRAF V600-Mutant Anaplastic Thyroid Cancer	25.76	2018
13	2021 American Thyroid Association Guidelines for Management of Patients with Anaplastic Thyroid Cancer	23.84	2021
14	Evaluation of Overall Survival in Patients with Anaplastic Thyroid Carcinoma, 2000-2019	17.54	2020
15	PD-1 Blockade in Anaplastic Thyroid Carcinoma	17.54	2020

Keywords can also assist us in efficiently identifying the prevalence and development of hotspots in the field of ATC therapies, besides references with citation bursts. The following keywords include expression, apoptosis, prognostic factors, activation, *in vitro*, BRAF, metastasis and doxorubicin, etc. after excluding keywords like thyroid cancer, tumors, thyroid, and carcinoma, etc. We came to the conclusion that the research on ATC treatments mostly focused on the following aspects, in accordance with keyword clustering analysis and trend topic analysis:

#### 4.3.1 Conventional treatments and new promising therapies

Many clinical case reports indicate that individuals who undergo surgical resection have a higher overall survival rate than those who have not undergone surgery when the limited lesion of ATC may be removed. However, Surgery is frequently limited in ATC due to the frequent presence of distant metastases. Thyroidectomy is an option if the cancer is restricted to the thyroid gland, particularly if the diameter of a single lesion is less than 5 cm and there is no evidence of dissemination. A complete or nearly total thyroidectomy in conjunction with therapeutic lymph node dissection in the neck’s central and lateral regions is the most beneficial surgical approach (32). Furthermore, research has demonstrated that total surgical resection of lesions (R0/R1) can improve patients’ overall and disease-free survival, regardless of the combination of adjuvant radiation and chemotherapy (36). External radiation therapy (XRT) is an important component of effective ATC treatment, whether it is used as part of comprehensive treatment after successful resection of the primary lesion or in active palliative treatment after incomplete or unresectable tumor resection. In patients with ATC whose lesion cannot be surgically removed entirely, radiotherapy can decrease tumor volume, lessen local complications, enhance surgical possibilities, and improve patient survival rates while also effectively lowering the risk of ATC metastasis and recurrence (32). Since ATC is frequently identified as a systemic disease that usually results in both distant and lymph node metastases. Another crucial component of systemic treatment is chemotherapy. For ATC patients with unresectable tumors and those in the advanced stage who plan active treatment, it is used as an initial and potential transitional method before emerging treatments such as targeted therapy and immunotherapy are feasible (32, 37).

As per the most recent guidelines issued by the American Thyroid Association (ATA), the recommended approach for managing non-resectable ATC involves employing primary systemic treatment modalities in the absence of molecular abnormalities. These treatment options include the administration of genotoxic drugs, such as a combination of paclitaxel and carboplatin, paclitaxel as a standalone therapy, or doxorubicin as a standalone therapy (3, 32). As a consequence of the inherent resistance to chemotherapy, targeted therapeutic approaches tend to be preferred when systemic treatment is necessary. The utilization of high-throughput sequencing techniques has revealed the molecular changes associated with ATC, therefore providing opportunities for the development of tailored therapeutic interventions (38–40). The understanding of molecular changes in patients with anaplastic thyroid carcinoma has gained increasing significance in clinical practice, particularly due to the emergence of targeted therapeutic approaches. Within the field of ATCs, the *BRAF V600E* mutant appears to be the prevailing and clinically relevant genetic alteration, observed in around 50–70% of cases (38). With the recent approval of a combination therapy combining the BRAF inhibitor dabrafenib and the MEK inhibitor trametinib for patients with metastatic or unresectable *BRAFV 600E*-positive ATC, several clinical trials have provided evidence about the effectiveness and safety of dabrafenib in conjunction with trametinib for managing ATC (23, 41, 42). Furthermore, certain pharmaceuticals, such as TKI inhibitors, RET inhibitors, mTOR inhibitors, CDK4/6 inhibitors, and antiangiogenic medicines, present significant therapeutic possibilities (43–47).

In recent decades, the field of anti-cancer therapy has been significantly transformed by the emergence of immuno-oncologic medications, particularly immune checkpoint inhibitors (ICI). The exploration of immunotherapy for individuals identified as having anaplastic thyroid cancer is currently ongoing. The expression of *CD274*, which encodes programmed death 1 (PD-L1), a ligand for the immunological checkpoint protein PD-1, has been observed to be increased in ATCs, proposing novel therapeutic options for ATC through the utilization of immunotherapy (48). The efficacy of Spartalizumab, an immunotherapy medication that targets the PD-1 pathway, has been investigated in the context of ATC. The results from the phase 2 segment of this trial indicated a response rate of 19%. The overall group exhibited a median OS of 5.9 months, with a one-year survival rate of 40% among patients. The median progression-free survival (PFS) observed in the study was 1.7 months (49). It is noteworthy that individuals exhibiting PD-L1 expression levels below 1% demonstrated a median OS of 1.6 months, with no observed responses within this subgroup. Conversely, patients with PD-L1 expression levels ranging from 1% to 49% and those with expression levels of 50% or higher exhibited a median OS that has not yet been determined, along with overall response rates of 18% and 35%, respectively. The subset of patients with a PD-L1 expression level greater than 50% exhibited the greatest observed rate of response, which amounted to 35%. Nevertheless, it should be noted that Spartalizumab has not received approval from the U.S. Food and Drug Administration (FDA) and is currently unavailable for commercial use. However, the outcomes derived from clinical trials, including the utilization of immunotherapy as a standalone treatment, have demonstrated restricted effectiveness. The efficacy of combining PD-1/PD-L1 immunotherapies with various kinase inhibitors has been demonstrated in the management of ATC (50, 51). Additional research is warranted in the near future to explore other options beyond the use of anti-cytotoxic T-lymphocyte antigen 4 (CTLA-4)/PD-1/PD-L1 antibodies, validate predictive biomarkers, and improve the selection of research communities for clinical trials in ATC (52).

#### 4.3.2 Mechanism

There was an expanse of many areas of research exhibited in **Figure 9**. For the purpose of identifying the ATC mechanism, issues concerning the mechanisms of growth, migration, apoptosis, inhibition, and differentiation were highlighted in basic medical trials. Long-standing goiters or other differentiated thyroid tumors might be the origin of ATC (53). Dedifferentiation is a complex process that occurs throughout the transition from DTC to PDTC and then to ATC, and is linked to stemness-like behavior and an increased risk of metastasis (54). Dedifferentiation is characterized by a number of genetic, epigenetic, and molecular events, such as chromosomal change (gains, deletions, and rearrangements.), genetic mutations (*BRAF, RAS, TERT*, and *P53*, etc.), and dysregulation of a number of signaling pathways (MAPK, PI3K/AKT/mTOR, NF-B, etc.), even though the exact mechanism underlying this phenomenon is unknown (55). The occurrence of *BRAF* mutations, *TERT* promoter mutations and *TP53* mutations are the most prevalent genetic alterations observed in ATC (14). The findings from *in vitro* experiments demonstrated that the inhibition of PATZ1 in thyroid cancer cells resulted in a decrease in the biological activity of p53, hence promoting both epithelial-mesenchymal transition (EMT) and cellular migration. Additionally, the restoration of PATZ1 initiated the activation of the p53 signaling pathway, resulting in a notable decrease in cellular migration and invasion (56). Some researchers have shown evidence for the widespread occurrence of mutations in the *TERT* promoter (specifically the *C228T* and *C250T*) in ATC. These findings indicate these mutations play a crucial role in conferring immortality and promoting aggressive behavior in ATCs. The association between the *C228T* mutation in the *TERT* promoter and the occurrence of distant metastases in ATC has been demonstrated. Furthermore, it is worth noting that the prognosis for individuals with ATC who had *TERT* promoter mutations, together with *BRAF* or *Ras* mutations, is exceedingly poor (14, 57). The complicated molecular process of ATC was investigated by Liu and colleagues through a thorough analysis of mRNA and miRNA expression profiles. Their studies have demonstrated that the upregulation of *CTHRC1, VCAN*, and *POSTN* plays a pivotal role in the initiation of angiogenesis, hence facilitating the provision of essential nutrients to tumor cells. Subsequently, the initiation of distant metastasis in the tumor is facilitated through the activation of cell migration and proliferation, as well as the modulation of intercellular connections. Furthermore, alterations in the intracellular concentration of potassium indirectly facilitate the advancement of ATC (58). ATC displays heterogeneity both between and within tumors, posing challenges in adequately characterizing its etiology. The progressive acquisition of diverse mutations throughout the carcinogenesis process results in the development of infinite proliferative capacity, enhanced metastatic potential, and resistance to several therapeutic interventions. The etiology of ATC has received relatively less research focus compared to other types of thyroid malignancies. Therefore, there is a need for additional investigation to elucidate the molecular mechanisms underlying the progression of ATC. Furthermore, it is crucial to identify distinct molecular subtypes that can provide valuable insights for guiding therapeutic strategies in ATC management. Despite the lack of clarity regarding the precise process and development of this phenomenon, experts from many global regions continue to engage in ongoing exploration and investigation. In the foreseeable future, there is a strong likelihood that our comprehension of the molecular mechanisms that drive the pathogenesis of anaplastic thyroid carcinoma will experience substantial advancements. These advancements have the potential to facilitate the emergence of novel therapeutic options that may prove advantageous.

#### 4.3.3 Prognostic factors

ATC responds poorly to standard therapy when compared to other cancers, necessitating multidisciplinary combination therapy. The prognosis for ATC is still awful and 80% of patients pass away within a year of their diagnosis, even though a variety of therapeutic methods are extensively used (32). ATC sufferers are more inclined to die early since early diagnosis is difficult and treatment fails to treat them effectively. Therefore, identifying the independent risk factors is crucial in order to apply prognostic categorization and individualized management. Based on the Surveillance, Epidemiology, and End Results (SEER) data, Cui et al. analyzed independent risk variables associated with early ATC death in the ATC population. This study identified specific risk factors for all-cause early death and cancer-specific early death, such as gender, tumor size, M-stage, surgery, radiation, and chemotherapy. Early intervention and rescue can be applied to patients based on these risk factors to increase their possibility of survival (59).

A 20-year retrospective study from a single Chinese medical facility assessed the benefits of multimodal therapy in raising the OS rate of ATC patients. In this study, the target population was separated into two subgroups: one group underwent surgery alone, and the other one underwent surgery along with radiotherapy and medication therapy (including chemotherapy, targeted therapy, and immunotherapy). The results of the research indicated that involvement of the recurrent laryngeal nerve, distant metastases, increased white blood cells, and treatment approaches were all independent risk variables for OS in univariate survival analysis. Multimodal therapy significantly improved OS when compared to single surgery. Multiple treatments were capable of helping to improve prognosis, and normal white blood cell counts and no distant metastases at initial diagnosis were independent protective factors for OS (60). Along with the clinical characteristics that affect prognosis listed above, molecular biomarkers for the prognosis of ATC, including gene mutation profiles, epigenetic profiles, microRNA profiles, and cancer stem cell markers, are also being intensively explored (61). The prognosis of ATC patients should be enhanced by novel intervention modalities developed for these prospective prognostic markers (such as small-molecule targeted medicines, new immunotherapies, etc.), which could also enhance survival time and quality.

## 5 Limitations

The present work used three bibliometric approaches together to analyze the pertinent literature on ATC treatment that was pulled from the WoSCC database, although it still has significant drawbacks. First, the data were sourced exclusively from a singular database, hence disregarding other databases that may contain relevant data. Additionally, our study focused exclusively on research published in the English language, perhaps leading to an underestimation of non-English-written publications. Finally, it ought to be noted that while bibliometrix has its merits, it cannot entirely substitute for systematic searching. Therefore, for a more precise literature analysis, it is advisable to utilize software analysis in conjunction with specific literature to develop a comprehensive analysis. Consequently, this study may exhibit inherent bias, perhaps leading to less complete and precise conclusions.

## 6 Conclusions

Anaplastic thyroid carcinoma is classified as an endocrine tumor characterized by its elevated aggressiveness, unfavorable prognosis, and the absence of currently viable therapeutic interventions. Hence, it is imperative to undertake a thorough bibliometric analysis of the subject of ATC treatment. This analysis entailed an extensive bibliometric review encompassing growth trends, prominent countries and institutions, core journals, co-citation analysis, and other important aspects. Several scientific visualization maps have been produced to illustrate the potential of research on ATC treatment. The exponential growth in the quantity of papers serves as evidence that the field of ATC treatment is garnering escalating scholarly interest on a global scale. The United States predominantly represents the top nations in this particular study domain. However, there is a need to enhance the collaborative and communicative frameworks across countries. The putative mechanisms behind the occurrence and progression of ATC, as well as potential prognostic variables, are being subject to incremental investigation. Various therapeutic modalities, such as targeted therapy, immunotherapy, traditional therapy, and multimodal therapy, are currently being implemented in the field of medicine. Additionally, ongoing clinical trials are being conducted to further explore the efficacy and potential of these treatment approaches.

In conclusion, this study offered an objective and comprehensive assessment of pertinent literature pertaining to ATC treatment, thereby serving as a helpful guide for researchers in this particular domain.

## Data availability statement

The original contributions presented in the study are included in the article/supplementary material. Further inquiries can be directed to the corresponding authors.

## Author contributions

SL: Data curation, Investigation, Methodology, Writing – original draft. XY: Data curation, Methodology, Writing – original draft, Conceptualization, Formal analysis. YY: Conceptualization, Supervision, Writing – original draft, Writing – review & editing. YX: Conceptualization, Supervision, Writing – review & editing. PZ: Conceptualization, Supervision, Writing – review & editing.
